# Interaction of amyloid beta with humanin and acetylcholinesterase is modulated by ATP

**DOI:** 10.1002/2211-5463.13023

**Published:** 2020-11-18

**Authors:** Sarah Atali, Sadaf Dorandish, Jonathan Devos, Asana Williams, Deanna Price, Jaylen Taylor, Jeffrey Guthrie, Deborah Heyl, Hedeel Guy Evans

**Affiliations:** ^1^ Chemistry Department Eastern Michigan University Ypsilanti MI USA

**Keywords:** acetylcholinesterase, amyloid‐beta, humanin, kinetics, lung cancer, peptide interaction

## Abstract

Humanin (HN) is known to bind amyloid beta (Aβ)‐inducing cytoprotective effects, while binding of acetylcholinesterase (AChE) to Aβ increases its aggregation and cytotoxicity. Previously, we showed that binding of HN to Aβ blocks aggregation induced by AChE and that HN decreases but does not abolish Aβ‐AChE interactions in A549 cell media. Here, we set out to shed light on factors that modulate the interactions of Aβ with HN and AChE. We found that binding of either HN or AChE to Aβ is not affected by heparan sulfate, while ATP, thought to reduce misfolding of Aβ, weakened interactions between AChE and Aβ but strengthened those between Aβ and HN. Using media from either A549 or H1299 lung cancer cells, we observed that more HN was bound to Aβ upon addition of ATP, while levels of AChE in a complex with Aβ were decreased by ATP addition to A549 cell media. Exogenous addition of ATP to either A549 or H1299 cell media increased interactions of endogenous HN with Aβ to a comparable extent despite differences in AChE expression in the two cell lines, and this was correlated with decreased binding of exogenously added HN to Aβ. Treatment with exogenous ATP had no effect on cell viability under all conditions examined. Exogenously added ATP did not affect viability of cells treated with AChE‐immunodepleted media, and there was no apparent protection against the cytotoxicity resulting from immunodepletion of HN. Moreover, exogenously added ATP had no effect on the relative abundance of oligomer versus total Aβ in either cell line.

## Graphical abstract

Both HN and AChE can bind Aβ in the absence of added ATP. Addition of ATP increases the binding affinity of Aβ to HN but not to AChE.

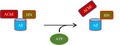

**Abbreviations**
AChE: acetylcholinesterase
AD: Alzheimer's disease
Aβ: amyloid beta
HN: Humanin
ID: immunodepletion

Besides its well‐documented intracellular role as a molecular energy source, ATP is known to be a ubiquitous extracellular messenger that acts on purinergic receptors to activate a number of intracellular signaling cascades [1, 2]. While the concentration of extracellular ATP in normal tissues has been found to be ~ 1–5 μm, it is elevated in the tumor microenvironment to levels (> 100 μm), which might induce normal cells to undergo apoptosis [1, 3]. Cancer cells that include non‐small‐cell lung carcinoma (NSCLC) A549 cells have been shown to release ATP and tolerate extracellular ATP concentrations that would otherwise lead to a cytotoxic response in normal cells [1].

Almost all types of cells produce amyloid beta (Aβ), a peptide well recognized for its role in the development and progression of different stages of Alzheimer's disease (AD) [4, 5, 6, 7, 8]. The Aβ peptide is ~ 4 kDa and derived from the sequential processing of the higher molecular weight amyloid precursor protein by two membrane‐bound endoproteases, β‐ and γ‐secretase [4, 9]. Different C‐terminal Aβ heterogeneity results from processing by γ‐secretase, where Aβ40 represents the most abundant isoform (~ 90%) as compared to Aβ42 (~ 10%) [4, 9]. Self‐assembled Aβ40/42 peptides into amyloid fibrils are thought to be implicated in the pathology of more than 20 devastating and serious human disorders, including AD and other neurodegenerative diseases [6, 7, 8, 10, 11, 12, 13]. The Aβ40 peptide has a lower tendency to form oligomers, shows lower aggregation kinetics, and displays lower toxicity than Aβ42 [7]. Of the two main forms of Aβ in the brains of patients with AD, Aβ42 has enhanced amyloidogenicity and is more toxic and fibrillogenic with faster aggregation kinetics [14]. The sequence of Aβ is partitioned into a hydrophilic N‐terminal region, while the C‐terminal part is composed of nearly all hydrophobic amino acids, proposed to account for its propensity to aggregate at neutral pH [15].

The mechanisms by which Aβ monomers are converted into functional entities and various types of dysfunctional assemblies are largely obscure [16]. Complementary approaches [17], employing molecular dynamics simulations and experimental methodology, have provided information about inhibitors that reduce aggregation and toxicity of different Aβ species [18, 19] and structural details of a broad range of interconverting Aβ assemblies that range in size, conformation, and toxicity between monomers [20, 21, 22, 23], oligomers [6, 24], protofibrils [25], and fibrils [26].

A rapidly growing body of evidence has recently steadily emerged showing that AD patients might have a reduced risk and some protection against certain cancers. [27] Inverse associations between cancer and AD [28, 29, 30, 31, 32, 33] have been reported showing that patients with AD generally had a significantly reduced risk of developing cancer with time, while individuals diagnosed with cancer have a reduced likelihood of living long enough to develop AD [27]. The incidence of AD was found to be reduced in glioblastoma and in other types of cancers including lung cancer [33]. Experimental evidence indicates that Aβ is protective against certain types of cancer and could inhibit the growth of tumor cells [34, 35]. Following treatment of cancer cell lines with conditioned media containing Aβ, proliferation of human breast adenocarcinoma, melanoma and glioblastoma [35] was inhibited. Direct injection of Aβ into human lung adenocarcinoma xenografts was also found to suppress tumor growth in mice [34]. Plasma levels of Aβ40 and Aβ42 were reported to be higher in all cancer patients compared with normal controls [36]. To gain further mechanistic insights into regulation of Aβ in lung cancer cells, we used two human NSCLC cell lines [37], A549 (p53‐positive) and H1299 (p53‐null) cells [38] in this study.

Soluble oligomers of proteins implicated in different diseases are primarily thought to be the main toxic form as opposed to the larger fibrillar assemblies [13, 39, 40]. Aβ regions containing Tyr and Ser (H_6_DSGY_10_ and G_25_SNKG_29_) (Fig. 1) along with post‐translational modifications of these regions, have been implicated in misfolding, oligomerization, or fibril formation of Aβ [5, 10, 11, 16, 41]. ATP is known to be protective against Aβ‐mediated cytotoxicity [42], and lower extracellular ATP levels were found to correlate with increased misfolded extracellular Aβ in AD [43, 44]. Aβ proteins are known to bind DNA and RNA [42, 45] with residues 25–35 comprising the DNA binding region. This region is within the GxxxG motif on Aβ (Fig. 1), involved in both Aβ oligomerization and nucleotide binding [42, 46]. Computational and biochemical studies demonstrated that ATP strongly interacts with both Tyr10 and Ser26 of Aβ fibrils (Fig. 1) and that both ATP and ADP reduced misfolding of Aβ at physiological intracellular concentrations, an effect that was enhanced by magnesium, the levels of which are known to be lowered in AD [42, 47]. In aqueous solution, monomeric Aβ is known to be intrinsically disordered but upon conversion into fibrils, amino acid residues Val12–Val24 and Ala30–Val40 each form a β‐strand with Gly25‐Gly29 forming a bend that results in parallel β‐sheets [41, 48]. Formation of this bent structure composed of Gly25‐Gly29 is thought to be an early event in self‐association of Aβ into fibrils [41, 48]. Asp23 was shown by computational studies to interact with Ser26, regulating the structure of Aβ [49], suggesting that phosphorylation of Ser26 may impact Aβ oligomerization and assembly.

**Fig. 1:**
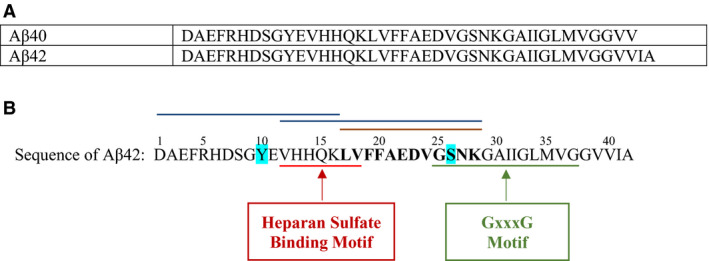
(A) Amino acid sequence of Aβ40 and Aβ42. (B) Regions of Aβ known to bind AChE (1–16, 12–28, blue lines) and HN (17–28, brown lines) are shown along with the heparan sulfate and GxxxG motifs. Tyr10 and Ser26 of Aβ previously reported to interact with ATP are shown in blue highlights.

A ~ 10^6^‐fold difference has been previously reported between the extracellular concentrations of ATP (nm‐low μm range) and the intracellular concentrations of ATP (mm range) [2, 42], a difference that was suggested to affect intracellular and extracellular folding of Aβ. Phosphorylation of Aβ was detected in primary cultures of mouse cortical neurons at low nanomolar concentrations of ATP, suggesting that Aβ can be phosphorylated *in vivo* at physiological concentrations of extracellular ATP [50]. In addition to ATP, the glycosaminoglycan, heparan sulfate (HS), has been previously reported to interact with Aβ peptides, increasing their aggregation [51, 52]. In both Aβ40/42, amino acid residues 12–18 (VHHQKLV) are reported to be important for interaction of Aβ with HS (Fig. 1).

Humanin (HN) is a secreted 21‐ to 24‐amino acid mitochondrial‐derived peptide [53, 54]. Certain amino acid residues in HN have been identified to be involved in different functions, including binding to Aβ [55, 56]. Growing evidence suggests that HN is a peptide with broad spectrum cyto‐ and neuroprotective actions that prevent different types of stress [55, 57, 58]. HN was identified previously as a binding partner of Aβ, likely modulating its aggregation pathways and counteracting its deleterious effects [56, 57, 59]. The morphology of Aβ40 was altered by HN from fibrillary to amorphous [60], likely protecting against Aβ‐induced cytotoxicity. Using circular dichroism and NMR [61], HN was found to be unstructured and flexible in aqueous solutions. HN was shown to take up a helical structure (Gly5 to Leu18) in a less polar environment, however, which might enable it to pass through membranes in its helical conformation forming specific interactions, while conformational changes leading to an unstructured form might allow the peptide to interact with different receptors [61].

Numerous attempts that employ a broad range of small molecule and peptide inhibitors are currently underway to delay the self‐assembly of monomeric Aβ into oligomeric forms [5, 13, 62]. While similar structures are adopted by Aβ40 and Aβ42 when part of the fibril, minimal information of the three‐dimensional structures of monomers and oligomers of either Aβ40 or Aβ42 in aqueous solution is currently available [6]. HN has been shown earlier to directly interact with Aβ oligomers [63]. Therefore, and due to its known function as a natural broad spectrum cytoprotective peptide, direct binding of HN to Aβ may enable it to block formation and/or toxicity of aggregated Aβ assemblies.

Amino acid residues involved in direct interactions between HN and Aβ40 were identified previously by molecular modeling [64]. The specific epitopes at the binding interface between HN and Aβ40 were identified, by proteolytic epitope excision and extraction in addition to affinity–mass spectrometric data analysis, to be HN (5–15) and Aβ (17–28) [64]. Binding of HN to Aβ (17–28) was suggested to block Aβ from interacting with its receptors [56]. Inhibiting the 17–28 region of Aβ reduced aggregation of the neurotoxic amyloid fibrils and related cytotoxicity in SH‐SY5Y, a human neuroblastoma cell line [8]. HN was also found to bind directly to Aβ42 and exhibit antioligomeric activity [63]. We also showed that Leu11 of HN is important for its binding with Aβ40 [65]. HN with a D‐isomerized Ser14 was found by NMR in an alcohol/water mixture solution to bind Aβ40 with greater affinity than either wild‐type HN or HNS14G, and possess strong inhibitory effects against Aβ40 fibrillation [66]. D‐isomerization of Ser14 led to a drastic conformational change in HN, an observation that might shed light on its cytoprotective molecular mechanism [66].

Acetylcholinesterase (AChE) is an enzyme known for its role in terminating acetylcholine‐mediated neurotransmission at the synaptic cleft [67]. The majority of the cortical AChE in the Alzheimer's brain is mainly associated with the amyloid core of senile plaques [68, 69, 70, 71, 72]. AChE forms a stable complex with Aβ during its assembly into filaments, increasing the aggregation and neurotoxicity of Aβ fibrils to levels higher than those of the Aβ aggregates alone [72, 73]. AChE increases Aβ42 oligomeric formation [74] and is known to be associated with amyloid plaque accumulation of abnormally folded Aβ40, considered as a main component of the amyloid plaques found in the brains of AD patients [67, 68, 69, 70, 71, 72, 73]. Addition of AChE significantly accelerated the aggregation of Aβ40 and assembly into Alzheimer's fibrils via decreasing the lag phase of the aggregation of the peptide, likely by a mechanism affecting the nucleation step and/or fibril elongation [68, 70, 71, 72, 73]. Noncatalytic functions of AChE were suggested earlier since the catalytic active center of the enzyme was not required for Aβ40 amyloid fibril formation [75]. The AChE peripheral anionic site was identified as the site where Aβ interacts, accelerating formation of amyloid fibrils and leading to a highly toxic complex [74]. Higher toxicity was associated with the AChE–amyloid complexes as compared to the toxicity of the Aβ aggregates alone [72]. Binding assays indicated [71] that AChE binds to Aβ (12–28), as well as to the Aβ (1–16) peptide (Fig. 1), directly promoting aggregation of Aβ40 and assembly into amyloid fibrils.

Access to the central domain of Aβ (residues 17–24) flanked by Lys‐16 and Lys‐28, known to be a critical structural element in fibrillar Aβ aggregates [76, 77, 78], might be regulated by binding of AChE and HN to their overlapping binding sites on Aβ. Despite the known opposing effects of HN and AChE on the oligomerization of Aβ [53, 74], we recently found that while the binding of AChE to Aβ is decreased in the presence of HN, it is not abolished, and that Aβ aggregation is greatly diminished in the presence of both HN and AChE to levels close to those induced by HN alone [79]. Moreover, we showed that the relative amount of Aβ oligomer versus total Aβ was increased upon immunodepletion (ID) of HN from A549 and H1299 lung cancer cell‐conditioned media, decreasing cell viability and increasing apoptosis [79].

Here, we set out to understand the effect of HS and ATP on the ability of Aβ to interact with either HN or AChE. We show that binding of HS does not alter the interaction of Aβ with either HN or AChE. However, upon addition of ATP, there was increased interaction between HN and Aβ but decreased affinity of Aβ for AChE. Moreover, exogenous ATP, added at concentrations that abolished AChE binding to Aβ in A549 cell media, did not protect against the cytotoxicity resulting from ID of HN in either A549 cells that express AChE or H1299 cells with minimal expression of the enzyme.

## Materials and methods

### Materials

Most of the material used was purchased as we previously reported [79, 80, 81]. Nitrocellulose membranes, PBS, ATP, recombinant human AChE (C1682, UniProt accession ID: C9JD78), streptavidin‐conjugated horseradish peroxidase (HRP) conjugate, Ponceau S solution, PMSF, and HS sodium salt from bovine kidney were purchased from Sigma‐Aldrich (St. Louis, MO, USA). Mouse IgG isotype control (mIgG), ultra 3,3′,5,5′‐tetramethylbenzidine (TMB)‐ELISA substrate solution, Halt Protease and Phosphatase Inhibitor Cocktail, HN polyclonal antibody (rabbit, PA1‐41610), and Nunc MaxiSorpTM 96‐well flat bottom plates were from Thermo Fisher (Waltham, MA, USA). Goat anti‐AChE antibody (ab31276) and rabbit anti‐Goat IgG H&L (HRP) (ab6741) were from Abcam (Cambridge, MA, USA). Mouse monoclonal amyloid‐β antibody (sc‐53822) and goat anti‐rabbit IgG‐HRP (sc‐2004) were from Santa Cruz Biotechnology (Dallas, TX, USA). The Super Signal West Pico Luminol (chemiluminescence) reagent and bicinchoninic acid protein assay kit were from Pierce (Waltham, MA, USA). Aβ40‐HFIP (AS‐64128‐05) and Aβ42‐HFIP (AS‐64129‐05), and biotin‐Aβ40 (AS‐23512‐01) and biotin‐Aβ42 (AS‐23523‐05) were purchased from AnaSpec (Fremont, CA, USA). AggreSure Aβ40 peptide (AnaSpec, AS‐72215) and AggreSure Aβ42 peptide (AnaSpec, AS‐72216) were purchased and pretreated to ensure that they are in a high % monomeric state. HN (018‐26) and biotin‐HN (B‐018‐26, UniProt accession ID: Q8IVG9) were purchased from Phoenix Pharmaceuticals (Burlingame, CA, USA). Anti‐Aβ antibody (6E10, 1–16, mouse), anti‐Aβ antibody (4G8, 17–24, mouse), anti‐Aβ42 antibody (mouse), and biotin anti‐Aβ antibody (4G8, 17–24) were from BioLegend (San Diego, CA, USA). Anti‐Aβ antibody (82E1, mouse) was purchased from IBL America (Spring Lake Park, MN, USA).

### Cell culture

Human NSCLC cell lines, A549 (ATCC CCL‐185) and H1299 (ATCC CRL‐5803), were purchased from the American Type Culture Collection (ATCC, Manassas, VA, USA). Cells were seeded as we reported earlier [80] in 5 mL HyClone Dulbecco's modified Eagle's medium/nutrient mixture F‐12 (DMEM/F12) (GE Healthcare Life Sciences, Pittsburgh, PA, USA), supplemented with 10% Fetalgro bovine growth serum (FBS; RMBIO, Missoula, MT, USA), 50 U·mL^−1^ penicillin, and 50 U·mL^−1^ streptomycin (Invitrogen, Life Technologies, Carlsbad, CA, USA) in 25‐cm^2^ tissue culture flasks, and allowed to grow overnight in an incubator at 37 °C, 95% humidity, and 5% CO_2_. The cells were counted after trypan blue staining, with a hemocytometer.

### ELISA

ELISAs were carried out as we previously reported [65, 80, 82]. Nunc MaxiSorp 96‐well flat bottom plate (Thermo Fisher) wells were coated with samples as indicated. The plates were incubated overnight at 4 °C on a shaker to allow binding of the samples to the plate wells. After the incubation, the wells were washed 4× with TBST, filled with 400 µL blocking buffer (110 mm KCl, 5 mm NaHCO_3_, 5 mm MgCl_2_, 1 mm EGTA, 0.1 mm CaCl_2_, 20 mm HEPES, 1% BSA, pH 7.4), and incubated with shaking overnight at 4 °C. The wells were then washed 4× with TBST, and 100 µL of sample at the desired concentration was added to each well and incubated with shaking overnight at 4 °C. TBST was then used to wash the wells 4× before proceeding in one of two ways: (a) biotinylated samples were analyzed by adding 100 µL streptavidin–HRP conjugate in TBST (1 : 2500 dilution) to the samples followed by incubation for 3 h at RT on a shaker, or (b) samples without biotin were analyzed by adding 100 µL TBST containing the primary antibody as per the manufacturer's recommendation, incubating for 3 h at RT on a shaker, followed by washing the wells 4× with TBST. The secondary antibody in 100 µL TBST was then added to the samples following the manufacturer's recommendation and incubated for 1 h at RT on a shaker. Plates containing either biotinylated or nonbiotinylated samples were then washed 5× with TBST followed by the addition of 100 µL TMB, which resulted in a blue color change. The reaction was stopped with 100 µL 2 m H_2_SO_4_ after incubating at RT for 0.5–15 min, resulting in a yellow color change, measured by absorbance at 450 nm. To monitor nonspecific binding, negative control wells on the plates included, for example, bound Aβ peptide then adding all components, streptavidin‐HRP and TMB, but without addition of biotin‐HN. Some wells were coated with 2.5, 10, 50, 100, 500, and 5000 nm biotin‐HN or Aβ to allow conversion of the OD measurements to concentrations of bound material. Before analysis, the OD from the data was corrected for nonspecific binding by subtracting the mean background absorbance for the negative controls. Typically, in control wells incubated on each plate, the background binding is about 10–15% of the maximum binding seen with addition of biotin peptides or antibodies. Statistical analysis was determined by the graphpad prism 8.4.3 software (San Diego, CA, USA). Data were expressed as the mean ± SD. Three independent experiments were carried out in triplicate for each assay condition.

### Quantitation of Aβ

Aβ ELISAs were carried out according to previous protocols [83, 84] for determining the oligomeric and monomeric concentrations of Aβ and as we recently reported [79]. Briefly, total Aβ (monomers + oligomers) was measured by two‐site binding ELISAs using the capture 6E10 monoclonal antibody and 4G8‐conjugated biotin as the detection antibody, which recognizes a distinct epitope, then quantitated using streptavidin‐HRP.

Using the same samples, oligomerized Aβ was measured by a single‐site ELISA in which antibodies targeting the same primary sequence epitope were used for both capture (4G8) and detection (4G8‐biotin). Only oligomers are detected with this approach since the 4G8‐biotin antibody cannot bind to the captured monomer because the epitope is blocked by the 4G8 capture antibody. Therefore, only oligomeric or multimeric Aβ containing additional exposed 4G8 epitopes, not engaged by the capture antibody, are reported by the streptavidin–HRP. The amount of the monomer was then estimated as the difference between the concentration of total Aβ and the concentration of the oligomer.

### MTT assay

The 3‐(4,5‐dimethylthiazol‐2‐yl)‐2,5‐diphenyl‐tetrazolium bromide (MTT) reduction assay (Sigma‐Aldrich), used to measure cell viability, was carried out as we reported earlier [80, 82, 85]. Cells were seeded in 96‐well plates as indicated in 200 μL 10% FBS‐supplemented media per well and maintained overnight at 95% humidity and 5% CO_2_. After an overnight incubation, the media were replaced with 200 μL serum‐free media, and the cells were further incubated, without or with different treatments, for 24, 48, or 72 h. The final concentration of DMSO in each well never exceeded 0.1%. The cells were then incubated for 4 h with MTT (0.5 mg·mL^−1^) in the dark. The media were carefully removed, and DMSO (100 μL) was added to dissolve the formazan crystals. The absorbance was measured at 570 nm in a plate reader. Untreated cells or wells containing only DMSO and media were used as a positive and negative control, respectively. Statistical analysis was conducted using graphpad prism version 8.4.3 for Windows. Significant values were considered at *P* < 0.05 and more significant values at *P* < 0.01, compared with the control.

### Immunodepletion

Conditioned media were immunodepleted (ID) according to the methods previously described [86] and our recently published report [79]. Briefly, specific antibodies were bound to ELISA wells overnight (1 : 1000 dilution). The wells were then blocked and washed, then 300 μL of the conditioned medium (0.5 μg·μL^−1^) treated as indicated, 72 h postserum starvation, was incubated with the antibodies bound to ELISA wells for 24 h. The ID media were then carefully removed and analyzed for the presence of the target protein or peptide by ELISA. The same amount of protein (3 µL of 600 µg·mL^−1^ total protein) of each sample was analyzed in the experiments. Significant depletion (95–100%) was observed upon using each of the antibodies employed in this study.

### Statistical analysis

The analysis was carried out as we previously reported [79, 81, 82]. Each experiment in this study was performed in triplicate and repeated a minimum of three times. Statistical values are expressed as the mean ± SD. To evaluate the statistical differences, the Mann–Whitney or Kruskal–Wallis (ANOVA) tests were used. All the statistical tests were two‐sided and a *P* value of < 0.05 was considered statistically significant in all cases. graphpad prism (GraphPad Software, 8.4.3) was used for the statistical analysis.

## Results and Discussion

### Binding of Aβ to either HN or AChE is not affected by HS

Humanin has been previously reported to bind amino acid residues 17–28 of Aβ (Fig. 1) [56, 57, 63, 64, 65, 87]. AChE is also known to bind Aβ (1–16) and the Aβ (12–28) region (Fig. 1) [10, 68, 70, 71, 74]. Since the binding sites of AChE and HN on Aβ are overlapping, we previously investigated whether HN and AChE can simultaneously bind Aβ [79]. We found that HN and AChE can each bind either Aβ40 or Aβ42 with comparable affinities [79]. Contrary to our expectations, however, we found that while binding of HN to Aβ decreased AChE‐Aβ interactions, both HN and AChE can simultaneously bind Aβ despite binding a shared sequence on the peptide (Fig. 1) [79]. To better understand the interactions of HN and AChE with Aβ, we tested their binding in the presence of the glycosaminoglycan, HS, reported earlier to interact with amino acid residues 12–18 (VHHQKLV) of Aβ40/42 peptides (Fig. 1) [51].

**Fig. 2:**
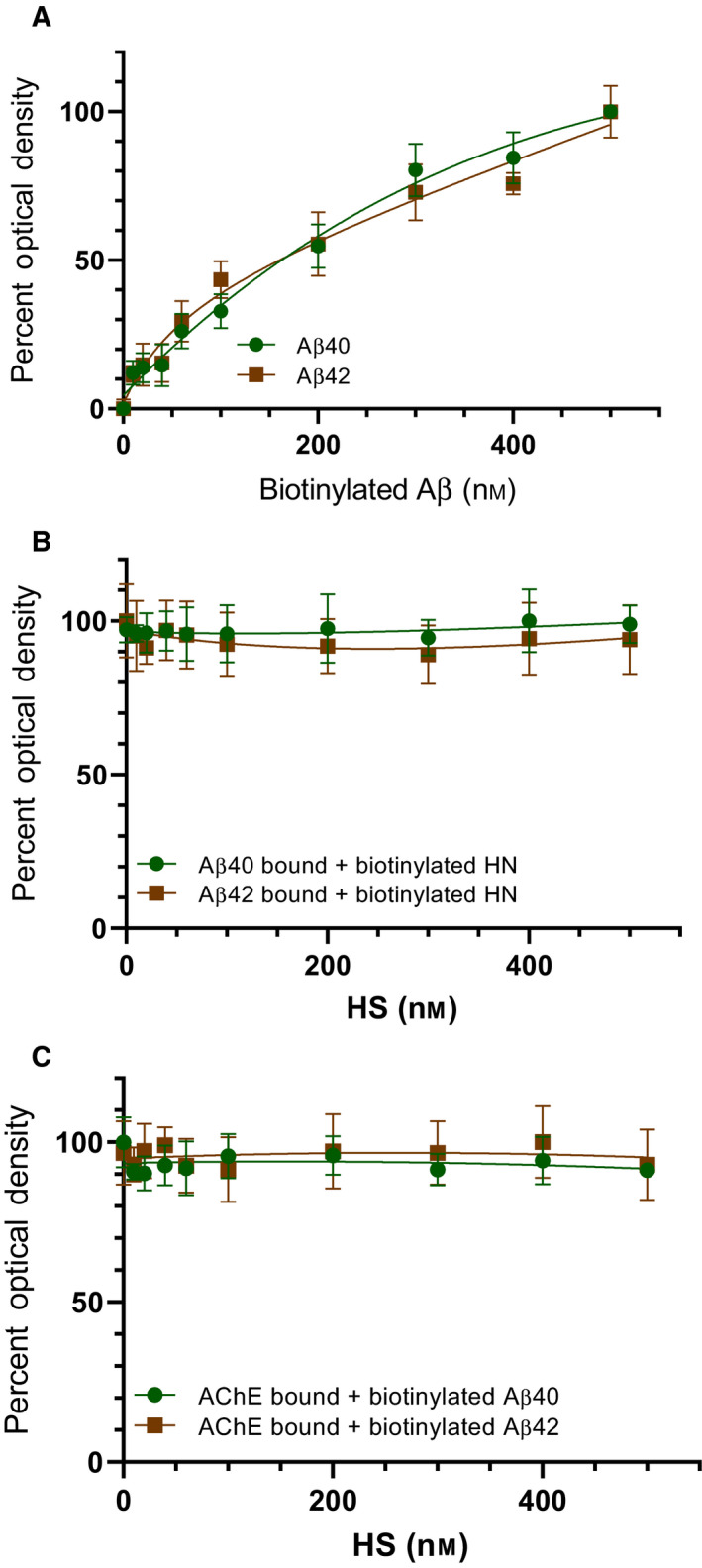
HS does not alter the binding of Aβ to either HN or AChE. (A) HS (100 nm) was bound to ELISA plate wells. Increasing concentrations of biotinylated‐Aβ were added to the wells and processed as described in Materials and methods. Optical densities (450 nm) were normalized for both curves by expressing each point relative to the best‐fitted *E*_max_ value (set to 100%). The data were then plotted as a function of increasing biotinylated‐Aβ concentrations and, using the graphpad prism 8.4.3 software, fit to a single binding site model with a nonlinear regression curve fitting approach. Data were expressed as the mean ± SD of three independent experiments, each carried out in triplicate. (B) Aβ (100 nm) was bound to ELISA plate wells. Biotinylated‐HN (300 nm) was then added to the wells in the absence or presence of increasing concentrations of HS. (C) AChE (10 nm) was bound to the wells. Biotinylated‐Aβ (1 μm) was then added in the absence or presence of increasing concentrations of HS. The negative controls had the same HN or Aβ and AChE concentrations, but water was substituted in place of biotinylated‐Aβ or biotinylated‐HN. Data were processed using the graphpad prism 8.4.3 software and presented as the mean ± SD of three independent assays.

Our previously published reports show that biotinylated‐Aβ interacts with the same affinity as the nonbiotinylated peptide with HN, and similarly, the binding of either HN or biotinylated‐HN to Aβ is indistinguishable [65, 79]. To examine the binding of Aβ40 and Aβ42 to HS, ELISA plate wells were coated with HS (100 nm). Increasing concentrations of biotinylated‐Aβ were then added (Fig. 2A) to the wells and processed as described in the Materials and methods. Optical densities (450 nm) were normalized for both curves by expressing each point relative to the best‐fitted *E*_max_ value (set to 100%). The data were plotted as a function of increasing biotinylated‐Aβ concentrations and fit to a single binding site model with a nonlinear regression curve fitting approach, using the graphpad prism 8.4.3 software. Both Aβ40/42 were found to bind HS with comparable affinities (Fig. 2A). We next tested whether increasing concentrations of HS can compete with binding of Aβ to either HN (Fig. 2B) or AChE (Fig. 2C). Aβ (100 nm) was bound to ELISA plate wells. Biotinylated‐HN (300 nm) was then added to the wells in the absence or presence of increasing concentrations of HS (Fig. 2B). Similarly, AChE (10 nm) was bound to ELISA wells followed by the addition of biotinylated‐Aβ (1 μm) in the absence or presence of increasing concentrations of HS (Fig. 2C). The negative controls had the same HN or Aβ and AChE concentrations, but water was substituted in place of biotinylated‐Aβ or biotinylated‐HN. Data were processed using the graphpad prism 8.4.3 software and presented as the mean ± SD of three independent assays. No effects of HS on the binding of Aβ40 or Aβ42 to either HN (Fig. 2B) or AChE (Fig. 2C) were observed at any of the HS concentrations used.

### ATP weakens interactions between AChE and Aβ but strengthens those between Aβ and HN

Aβ peptides are known to bind DNA and RNA [42, 45] with amino acid residues 25–35 comprising the DNA binding region found within the Aβ GxxxG motif (Fig. 1), involved in both Aβ oligomerization and nucleotide binding [42, 46]. ATP was shown to strongly interact with both Tyr10 and Ser26 of Aβ fibrils (Fig. 1) and reduce misfolding and fibrillation of Aβ at physiological intracellular concentrations, an effect that was enhanced by magnesium [42, 47]. Moreover, the aggregation of Aβ16–22 was reported, by an in silico study, to be highly unfavorable in the presence of ATP that formed hydrogen bonding, π–π stacking, and NH−π interactions with the Aβ16–22 peptide, preventing its aggregation [88].

**Fig. 3:**
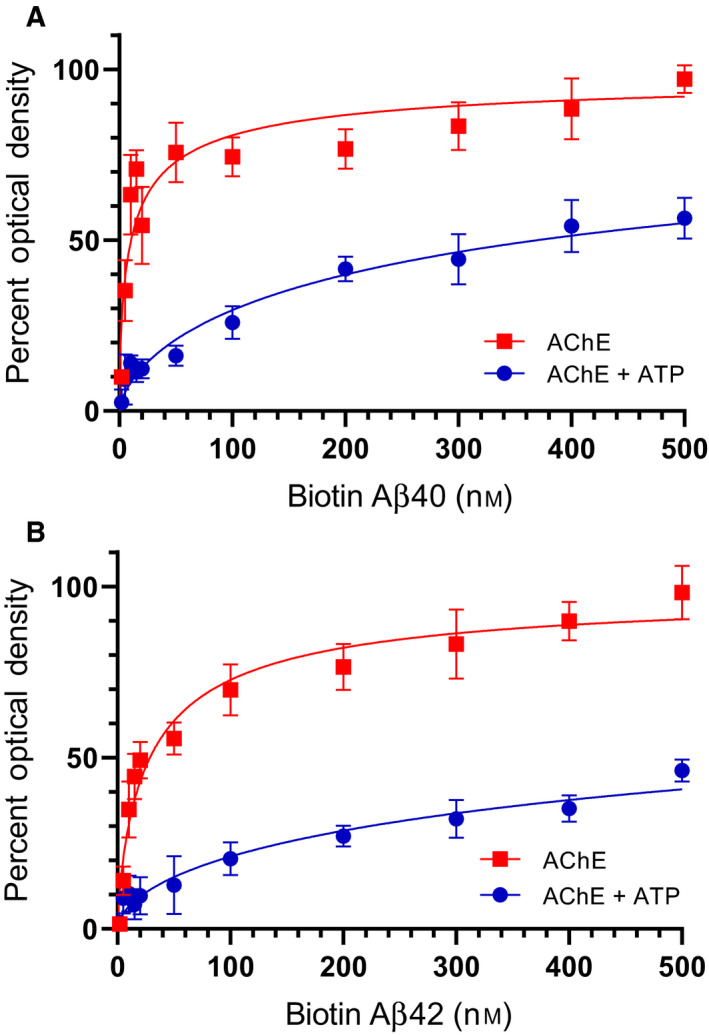
ATP diminishes the binding of AChE to Aβ. AChE (10 nm) was bound to the wells and then increasing concentrations of biotinylated‐Aβ40 (A) or biotinylated‐Aβ42 (B), in the absence or presence of 200 μm ATP, were added to the wells and processed as described in Materials and methods. Optical density measurements (450 nm) were normalized by expressing each point in relation to the best‐fitted *E*_max_ value (set to 100%) and plotted as a function of increasing biotinylated‐Aβ concentrations. The data were fit to a single binding site model with a nonlinear regression curve fitting approach using graphpad prism 8.4.3. Data were expressed as the mean ± SD of three independent experiments, each run in triplicate.

**Fig. 4:**
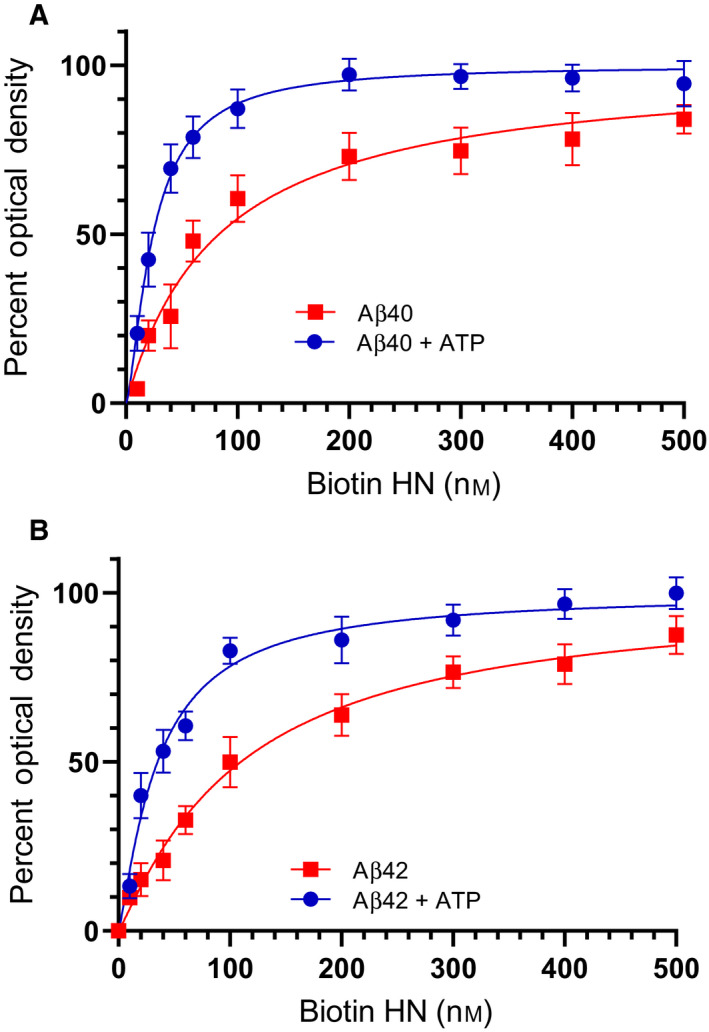
ATP increases the binding of HN to Aβ. Aβ40 (A, 100 nm) or Aβ42 (B, 100 nm), preincubated in a buffer in the absence or presence of 200 μm ATP, was bound to the wells, and then increasing concentrations of biotinylated‐HN were added and processed as described in Materials and methods. Optical density measurements (450 nm) were normalized by expressing each point in relation to the best‐fitted *E*_max_ value (set to 100%) and then plotted as a function of increasing biotinylated‐HN concentrations. The data were fit to a single binding site model with a nonlinear regression curve fitting approach using graphpad prism 8.4.3. Data were expressed as the mean ± SD of three independent experiments, each performed in triplicate.

To examine the effect of added ATP, if any, on the binding of either AChE or HN to Aβ, AChE (10 nm) was bound to ELISA plate wells (Fig. 3). Increasing concentrations of biotinylated‐Aβ, preincubated for 30 min at RT in 50 mm Tris/HCl, pH 7.5, 0.1 mm EDTA, 10 mm MgCl_2_, 2 mm DTT buffer without or with 200 μm ATP, were added to the wells and processed as described in the Materials and methods. Similarly, Aβ (100 nm) preincubated for 30 min at RT in 50 mm Tris/HCl, pH 7.5, 0.1 mm EDTA, 10 mm MgCl_2_, 2 mm DTT buffer without or with 200 μm ATP was bound to the wells (Fig. 4), and then, increasing concentrations of biotinylated‐HN were added to the wells and processed. Optical density measurements (450 nm) were normalized by expressing each point in relation to the best‐fitted *E*_max_ value (set to 100%) and then plotted as a function of increasing biotinylated‐Aβ or biotinylated‐HN concentrations. The data were fit to a single binding site model with a nonlinear regression curve fitting approach using the graphpad prism 8.4.3 software.

Addition of ATP reduced the affinity of AChE to both Aβ40 and Aβ42 (Fig. 3). Conversely, the affinity of either Aβ40 or Aβ42 for HN was increased (Fig. 4). The concentration of ATP used (200 μm) was chosen to be within the extracellular physiological concentrations previously found (> 100 μm) in the lung cancer cell lines used in this study [1, 2, 3]. These results might suggest that ATP binding to Aβ promotes optimal alignment of the amino acids needed to interact with HN increasing binding affinity, while conversely, it renders the amino acid residues on Aβ important for binding AChE, less accessible.

### More HN is found in a complex with Aβ upon addition of ATP to the conditioned media of either A549 or H1299 cells, while levels of AChE found in a complex with Aβ are decreased by ATP addition to A549 cell media

**Fig. 5:**
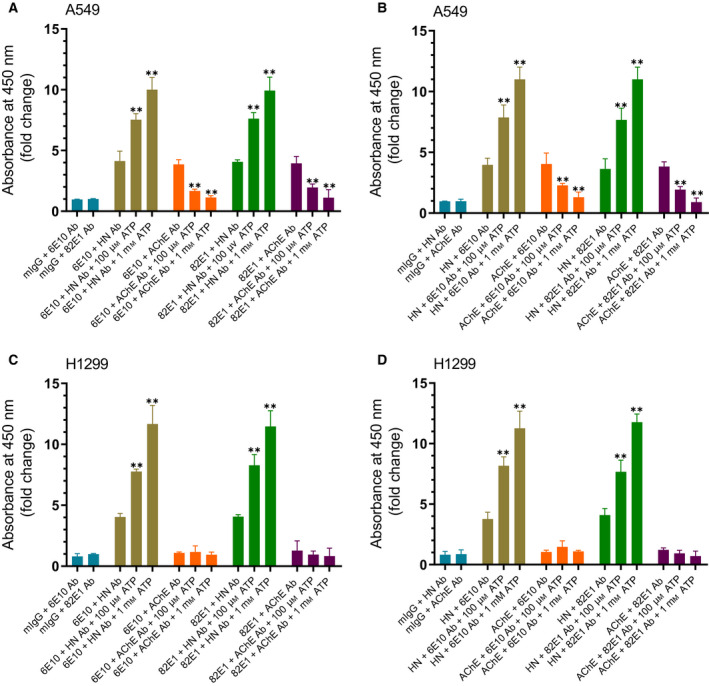
ATP increases the amount of HN found in a complex with Aβ in both A549 and H1299 cell media while AChE found in a complex with Aβ is decreased by the addition of ATP to A549 cell media. Cells (0.2 × 10^5^ cells per well) were seeded in 96‐well plates in 10% FBS‐supplemented media. The next day, the cells were incubated in serum‐free medium for 72 h. Specific antibodies were added (1 : 1000 dilution) to ELISA wells. After blocking the wells, 300 μL of the A549 (A, B) or H1299 (C, D) cell‐conditioned medium (0.5 μg·μL^−1^), 72 h postserum starvation, was added. The proteins/peptides were detected using their corresponding primary antibodies and then processed as described in Materials and methods section. Each column represents the mean ± SD of three independent separate experiments, each performed in triplicate. Data processing was carried out using the graphpad 8.4.3 software. Asterisks (**) indicate a statistically significant difference between each treatment relative to samples without ATP. Absence of asterisks indicates no significance, Mann–Whitney test, **P < 0.01.

We next set out to examine whether addition of ATP modulates the binding of either HN or AChE to Aβ using the conditioned media from A549 cells and H1299 cells that we previously used to examine these interactions [79]. Cells (0.2 × 10^5^ cells per well) were seeded in 96‐well plates in 10% FBS‐supplemented media. The next day, the cells were incubated in serum‐free medium for 72 h. Specific antibodies were added (1 : 1000 dilution) to ELISA wells (Fig. 5). After blocking the wells, 300 μL of the A549 or H1299 cell‐conditioned medium (0.5 μg·μL^−1^), 72 h postserum starvation, was added in the absence or presence of ATP (100 μm, 1 mm). The proteins/peptides were detected using their corresponding primary antibodies and then processed as described in Materials and methods section. It was previously shown that the antibody, 6E10, is highly specific for nonphosphorylated Aβ, while the antibody, 82E1, detects both phosphorylated and nonphosphorylated peptides [50]. Since the objective of this experiment was to test the binding of HN and AChE to Aβ in the conditioned media without and with added ATP, both antibodies that recognize all species of Aβ without regard to conformation were used in the case of any possible complications that might occur due to phosphorylated Aβ, if any (Fig. 5). The 6E10 antibodies are known to react with monomers, oligomers, and fibrils of Aβ [89, 90] and recognize the N‐terminal hydrophilic sequence, amino acids 1–16 of Aβ. This epitope, previously reported to be exposed in Aβ aggregates [89], has been shown by a high‐resolution mapping approach, to be residues 4–10 [91]. The 82E1 monoclonal antibodies are known to be specific to the N terminus of Aβ and recognize residues 1–16 [50].

The effects on the binding of HN to Aβ were comparable using either 6E10 or 82E1 antibodies bound to ELISA wells (Fig. 5) incubated with the conditioned media of either A549 or H1299 cells. Upon addition of 100 μm ATP to the conditioned media of A549 cells, there was an ~ 1.85‐fold increase in the amount of HN bound to Aβ (Fig. 5A), a level that was further increased to ~ 2.5‐fold upon addition of 1 mm ATP, relative to samples without added ATP. Similar results were obtained upon binding of ELISA wells with anti‐HN antibodies and using either 6E10 or 82E1 to detect Aβ from A549 cell‐conditioned media (Fig. 5B). Increased levels of HN bound to Aβ in the presence of ATP were also comparable using H1299 cell‐conditioned media (Fig. 5C,D). Recently, using human lung carcinoma NSCLC cell lines [82], we found that treatment of A549 cells (p53‐positive) with p53 siRNA blocked AChE expression while no change in the levels of the enzyme was found with this siRNA treatment using H1299 cells with a p53‐null genotype due to a biallelic deletion of the TP53 gene [38]. Binding of AChE from the conditioned media of A549 cells to Aβ was comparable when either 6E10 or 82E1 was bound to the ELISA plate wells (Fig. 5A). The levels of AChE found in a complex with Aβ were decreased by ~ 2.3‐fold upon addition of 100 μm ATP, then decreased to almost blank levels upon addition of 1 mm ATP (Fig. 5A). Binding anti‐AChE antibodies to the plate wells also showed a comparable decrease in the levels of Aβ detected by either 6E10 or 82E1 antibodies, upon addition of ATP (Fig. 5B). No AChE was detected above background using H1299 cell‐conditioned media (Fig. 5C,D), consistent with our previous report showing negligible levels of AChE in H1299 cells relative to those in A549 cells [82]. Despite differences in AChE expression in the two cell lines, however, interaction of HN from the conditioned media of both cell lines with Aβ appears to be augmented by the addition of ATP to a comparable extent (Fig. 5). These results might suggest that increased interactions of HN with Aβ by ATP may not be modulated by AChE.

### Exogenously added ATP increased interactions of HN from A549 and H1299 cell‐conditioned media with Aβ and correlated with decreased binding of exogenously added HN, and less AChE in a complex with Aβ from A549 cell‐conditioned media

**Fig. 6:**
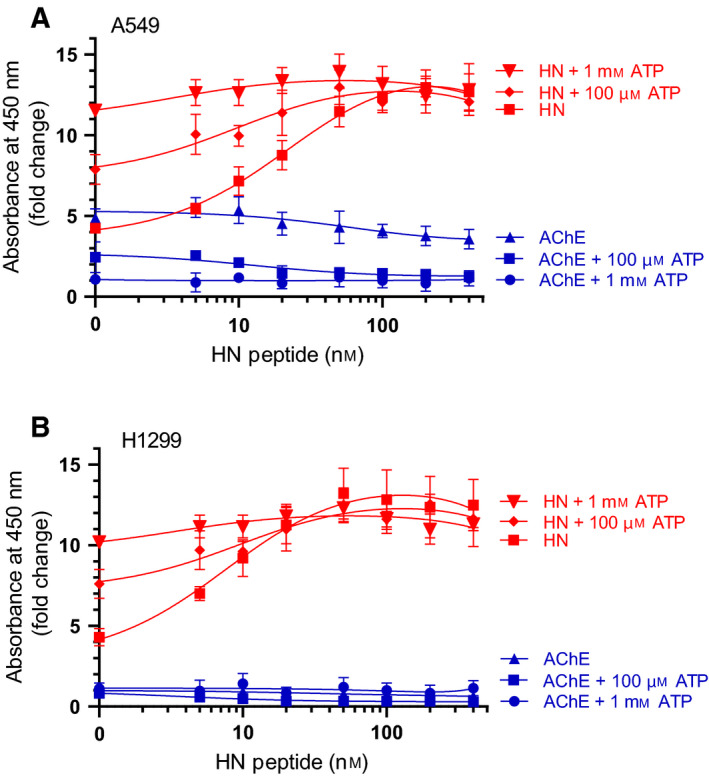
Exogenously added ATP increases HN bound to Aβ from media of A549 and H1299 cells using 82E1 antibodies, resulting in decreased binding of exogenously added HN to Aβ. Conversely, less AChE is found in a complex with Aβ in the presence of added ATP in A549 cells. Anti‐Aβ‐specific antibodies (82E1) were added (1 : 1000 dilution) to ELISA plate wells. The wells were blocked, and 300 µL of conditioned media (0.5 µg·µL^−1^) of A549 cells (A) or H1299 cells (B), 72 h after serum starvation, was added without or with ATP and increasing HN concentrations, and then, the HN and AChE bound were detected using the corresponding specific primary antibodies and processed as described in Materials and methods. Fold change relative to controls incubated with all components except the primary antibodies was calculated and fit, using the graphpad prism 8.4.3 software, with a nonlinear regression curve. The data represent the mean ± SD of three separate experiments, each performed in triplicate.

We previously reported that while HN weakens the interactions of AChE with Aβ, it does not abolish the enzyme's ability to bind Aβ [79]. Since our results showed (Fig. 5) that addition of ATP increases the interaction of HN with Aβ using the conditioned media from either A549 or H1299 cells but decreases the binding of AChE to Aβ in A549 cell media, we set out to understand how addition of ATP might affect binding of exogenously added HN to Aβ. Anti‐Aβ‐specific antibodies (82E1) were added (1 : 1000 dilution) to ELISA plate wells (Fig. 6). The wells were blocked, and then, 300 µL of the conditioned media (0.5 µg·µL^−1^) of A549 or H1299 cells, 72 h postserum starvation, was added in the absence or presence of increasing HN concentrations, and the HN and AChE bound were detected using the corresponding specific primary antibodies. Fold change relative to controls that included all components but without the primary antibodies was calculated and fit with a nonlinear regression curve using the graphpad prism 8.4.3 software.

While binding of AChE to Aβ immobilized to its antibody, 82E1, was detected using the A549 cell‐conditioned media, barely detectable levels of AChE from the conditioned media of H1299 cells were found to bind Aβ (Fig. 6). This finding is not surprising since we recently showed [82] that there are minimal levels of AChE in the conditioned media of the p53‐null cell line, H1299, as compared to the media from the p53‐positive cell line, A549. Binding of Aβ to AChE from the A549 cell‐conditioned media was decreased upon addition of ATP (Fig. 6A). In the conditioned media of both cell lines, HN was found bound to Aβ (Fig. 6). To examine the ability of exogenously added HN to bind Aβ upon addition of ATP, we incubated the wells with increasing concentrations of the HN peptide followed by washing the unbound material as described in Materials and methods. Addition of exogenous HN resulted in its increased binding to Aβ from both A549 and H1299 media bound to its immobilized antibody, 82E1. In both cases, increasing ATP concentrations resulted in increased binding of endogenous HN from the conditioned media and corresponded to decreased binding of exogenously added HN, an effect that was more pronounced using 1 mm as opposed to 0.1 mm ATP. These results likely indicate that the affinity of endogenous HN to Aβ is increased upon incubation with higher ATP concentrations in the media of both cell lines, which is expected to correlate with reduced binding of exogenously added HN. This interpretation is supported by the observed increased affinity of HN to either Aβ40 (Fig. 4A) or Aβ42 (Fig. 4B) in the presence of ATP. Moreover, the curves from each cell line treatment leveled off at comparable higher concentrations of exogenously added HN. The signal obtained upon adding HN increased, then began to level off around 50 nm (Fig. 6), concentration of HN was ~ 50 nm when measured previously [80] in the A549 media. Since the effect of added ATP on HN interactions with Aβ is comparable using either A549 or H1299 cell‐conditioned media and as AChE levels in H1299 media are negligible relative to those found in A549 cell media, one possibility is that AChE is inefficient at regulating the effects of ATP on the interaction of HN with Aβ. Our data show (Fig. 6) that the affinity of HN, added exogenously at lower concentrations, to Aβ is increased upon addition of ATP. The lack of an increase in the binding of HN, added exogenously at concentrations that exceed 50 nm in the absence or presence of ATP, suggests that binding of HN to Aβ is closer to reaching saturating levels under these conditions.

### Exogenously added ATP has no effect on A549 or H1299 cell viability

**Fig. 7:**
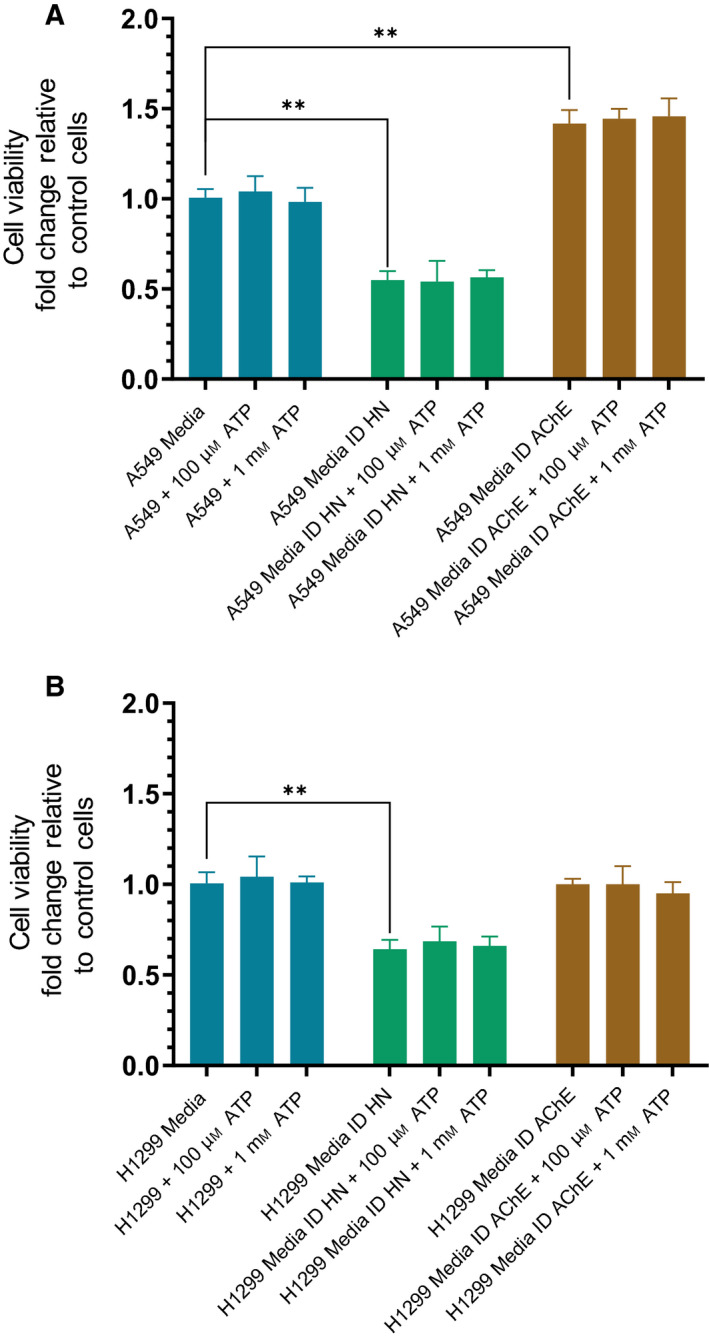
Addition of exogenous ATP has no effect on A549 or H1299 cell viability. Viability of A549 (A) or H1299 (B) cells was assessed by the MTT assay. Cells were seeded in 96‐well plates at 0.2 × 10^5^ cells per well in 200 µL 10% FBS‐supplemented media. The next day, the cell monolayers were incubated in serum‐free medium for 12 h, then treated with control media, HN or AChE‐ID media (Media ID), without or with ATP, for 48 h with the media containing the specific components in the various treatments replaced every 12 h. Data were processed using the graphpad 8.4.3 software. The graphs summarize the results expressed as means ± SD of three separate experiments, each performed in triplicate. Statistical differences between ID versus nondepleted media were analyzed by a one‐way analysis of variance (ANOVA) test, **P < 0.01.

Since addition of ATP appears to increase binding of HN to Aβ, and conversely, decreases the interaction of Aβ with AChE (Figs 3, 4, 5, 6), we tested the effect of added ATP on A549 and H1299 cell viability (Fig. 7). Exogenously added ATP at either 100 µm or 1 mm had no effect on viability of either A549 (Fig. 7A) or H1299 cells (Fig. 7B). These results are consistent with previous reports showing that there is no effect upon addition of either 100 μm or 1 mm ATP on A549 cell viability [3] suggesting that, compared with normal cells, lung cancer cells exhibit reduced cytotoxicity upon treatment with these extracellular ATP concentrations. Earlier, we found that ID of HN from A549 or H1299 cell‐conditioned media led to diminished cell viability [79]. To determine the effect of ID of AChE or HN in the absence or presence of added ATP on cell viability, ID media were prepared by first seeding 0.2 × 10^5^ cells per well in 96‐well plates in 10% FBS‐supplemented media. The next day, the cells were incubated in serum‐free medium for 72 h, then ID of AChE or HN as described in Materials and methods. For cell viability assays, cells were seeded in 96‐well plates at 0.2 × 10^5^ cells per well in 200 µL 10% FBS‐supplemented media followed by incubation in serum‐free medium for 12 h, then treated with the ID media for 48 h. The medium containing the specific components in the different treatments was replaced every 12 h. HN ID resulted in an approximate 0.45‐ and 0.35‐fold decrease in A549 and H1299 cell viability, respectively (Fig. 7). No change in cell viability was observed, however, upon treatment of A549 or H1299 cells with media ID of HN, with added ATP at either 100 µm or 1 mm concentrations as compared to that measured in the absence of added ATP (Fig. 7). Similarly, addition of ATP did not affect cell viability (Fig. 7) upon cell incubation with AChE‐depleted media. ID of AChE had no effect on H1299 cell viability (Fig. 7B) since they have minimal expression of the enzyme [82] while A549 cell viability increased (Fig. 7A) ~ 1.45‐fold upon AChE ID, effects that were not further modulated upon addition of ATP. This increase in cell viability upon ID of AChE from A549 cell media might reflect the recognized role of AChE as a tumor suppressor and a pro‐apoptotic gene in NSCLC cells that attenuates cell growth when its expression is upregulated [92, 93]. These tumor suppressor functions are known to be in part due to the catalytic hydrolysis of acetylcholine [92, 93, 94, 95]. Therefore, this increase in cell viability might reflect attenuating the general adverse effects of AChE on A549 cell viability that include its ability to induce Aβ aggregation.

These results indicate that differences in the binding of HN and AChE to Aβ upon addition of exogenous ATP have no impact on viability of either A549 or H1299 cells. Therefore, factors affecting cell viability appear to depend not on additionally added ATP or its consequent effects on modulating the binding of either HN or AChE to Aβ, but rather on depleting HN or AChE from the media. ATP is known to be protective against Aβ‐mediated cytotoxicity [42] and leads to reduced misfolding of Aβ at physiological intracellular concentrations [42, 47]. Reduction of extracellular ATP levels has been shown to correlate with increased misfolded extracellular Aβ in AD [43, 44]. In normal tissues, the concentration of extracellular ATP has been shown to be ~ 1–5 μm; however, in the tumor microenvironment, ATP levels rise to concentrations greater than 100 μm, amounts that might lead normal cells to undergo apoptosis [1, 3]. Cancer cells including NSCLC A549 cells have been found to release ATP and tolerate extracellular ATP concentrations that would otherwise lead to a cytotoxic response in normal cells [1]. Our results show that at the highest concentrations of ATP (1 mm) added extracellularly to A549 cell‐conditioned media, known to express higher levels of AChE as compared to H1299 cells [82], the levels of the enzyme bound to Aβ were close to blank values suggesting that the interaction of AChE with Aβ was blocked by addition of 1 mm ATP (Figs 5 and 6). Under these conditions, more HN was found bound to Aβ in both A549 and H1299 cells (Figs 5 and 6). However, addition of higher ATP concentrations does not seem to protect against the cytotoxicity resulting from ID of HN in either A549 or H1299 cells (Fig. 7) suggesting the need for further investigation into this observation.

### The relative amount of oligomer versus total Aβ upon immunodepletion of either AChE or HN from the cell‐conditioned media is unaffected by the addition of ATP

**Fig. 8:**
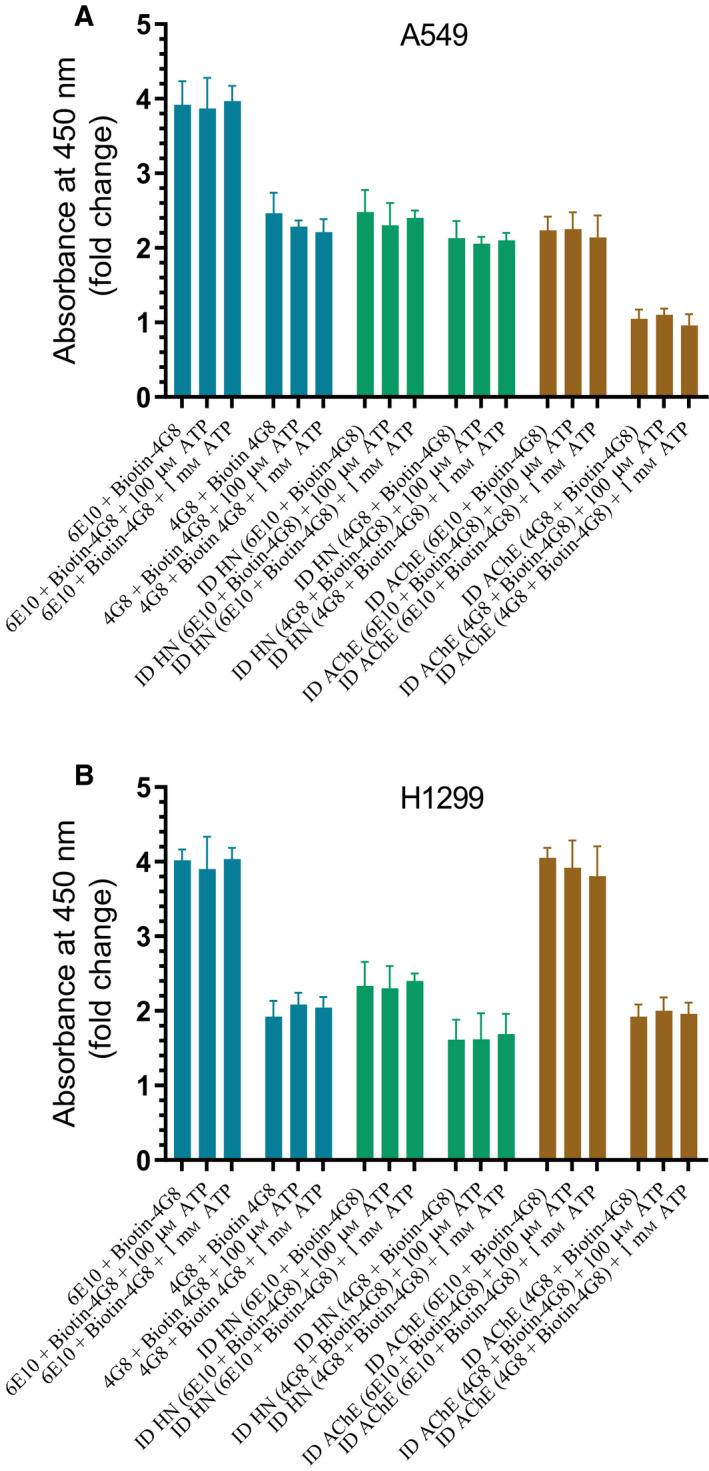
Addition of ATP does not affect the relative amount of oligomer versus total Aβ upon ID of HN from A549 (A) or H1299 (B) cell‐conditioned media. Cells (0.2 × 10^5^) were grown in 10% FBS‐supplemented media for 24 h. The cells were then incubated in serum‐free medium for 72 h without or with ATP and the media collected and ID from either HN or AChE as described in Materials and methods section. The antibodies 6E10 or 4G8 were bound (1 : 1000 dilution) to ELISA wells. The wells were blocked, and then incubated with 300 μL of the control and ID medium (0.5 μg·μL^−1^). Biotin‐4G8 was then added and the signal processed as described in Materials and methods section. Fold change relative to controls using anti‐6E10 and anti‐4G8 antibodies and 300 μL of the medium not incubated with cells was calculated. Data were processed using the graphpad 8.4.3 software. The graphs summarize the results expressed as means ± SD of three separate experiments, each performed in triplicate. Absence of asterisks indicates no significance compared to samples without added ATP, Mann–Whitney test.

Both epitopes recognized by the 6E10 and 4G8 antibodies have been previously shown to be exposed in Aβ aggregates [89, 96]. The 4G8 : 6E10 ratio was suggested to be a marker for the relative amount of aggregated versus monomeric Aβ [96]. To determine the effect of ID of AChE or HN on this ratio in the absence or presence of added ATP, 0.2 × 10^5^ cells per well were seeded in 96‐well plates in 10% FBS‐supplemented media. The next day, the cells were incubated in serum‐free medium for 72 h, then ID of AChE or HN as described in Materials and methods. The antibodies 6E10 or 4G8 were bound (1 : 1000 dilution) to ELISA wells (Fig. 8). The wells were blocked, and then incubated with 300 μL of the ID medium (0.5 μg·μL^−1^). Biotin‐4G8 was then added and the signal processed as described in Materials and methods section. Fold change relative to controls using anti‐6E10 or anti‐4G8 antibodies incubated with 300 μL of the medium not incubated with cells was calculated.

Relative to total Aβ, there was more oligomer in A549 cell‐conditioned media (~ 63%) as compared to that found in media of H1299 cells (~ 48%) (Fig. 8). ID of HN reduced the total Aβ in A549 cell media to ~ 63% and to ~ 58% in H1299 cell media. Compared to the total Aβ that remained after HN ID, there was relatively higher oligomer (~ 86% of total) in A549 cell media as compared to H1299 media (~ 69%). While those results might suggest that depletion of HN promotes the ability of AChE to increase Aβ oligomer formation in A549 cell media, this suggestion is unlikely to be correct since no change was found upon exogenously added ATP to A549 cell media (Fig. 8A), conditions under which the binding of the enzyme to Aβ was largely abolished (Figs 5 and 6). ID of AChE from A549 cell media decreased the total Aβ in the media to ~ 57% (Fig. 8A). The amount of the oligomer was ~ 47% of the total Aβ remaining after the ID of AChE suggesting that removing AChE from the A549 cell media reduces the ratio of oligomer to total Aβ relative to that in undepleted media. No change upon ID of AChE was observed in H1299 cell media (Fig. 8B), which is not surprising since expression of AChE is minimal in this cell line [82].

Our data show that the relative amount of oligomer versus total Aβ is increased upon ID of HN from A549 or H1299 cell‐conditioned media (Fig. 8) and correlates with diminished cell viability (Fig. 7). The mechanisms employed by HN in protecting against Aβ oligomerization or toxicity under these conditions are unclear. One can imagine, however, that depletion of HN renders Aβ susceptible to modifications increasing its oligomerization despite the higher concentrations of ATP. While binding of ATP might reduce misfolding of Aβ, the lack of effect found with exogenously added ATP on the relative amount of oligomer versus total Aβ (Fig. 8) using nondepleted media or media ID of either HN or AChE might indicate active processes occurring that use ATP to cause Aβ aggregation, leading to an overall negligible change in the oligomer versus total Aβ ratio. For example, various types of cancer cells, including lung adenocarcinoma, were reported to excrete extracellular cAMP‐dependent protein kinase [97], the activity of which is elevated, and in serum samples from patients with different types of cancers, constitutive kinase activity compared with normal, was reported. Extracellular Aβ was previously shown to be phosphorylated on Ser8 by protein kinase A that is either secreted or localized on the cell surface [98]. This phosphorylation, detected in transgenic mice and brains from human AD patients, promoted formation of toxic oligomeric and fibrillar Aβ assemblies both *in vitro* and *in vivo* [50, 98], and increased the stability of Aβ aggregates against dissociation into monomers by SDS [99]. In monomeric Aβ, Ser8 was found in a region of high conformational flexibility that upon phosphorylation undergoes structural changes favoring a less compact conformation in the N‐terminal region of Aβ present in insoluble aggregates [100]. In addition, unphosphorylated or Ser8 phosphorylated monomeric Aβ remained largely unstructured and disordered [100]. Besides Ser8, Aβ can also be phosphorylated on Ser26 by cdc2 or CK1 or nitrated on Tyr10 [50, 98, 101, 102]. Cdk1/cdc2 expression was shown to be upregulated in lung adenocarcinoma and correlated directly with the pathological clinical features and poor prognosis of the disease [103]. Depletion of cdk1 was found to slow G2‐M progression in the H1299 cell line [104]. Aβ Ser26 phosphorylation was reported to result in the formation and stability of the soluble oligomeric assembly of the peptide without further formation of larger prefibrillar or fibrillar aggregates, increasing neurotoxicity [39]. While nonphosphorylated Aβ and pSer8 Aβ were both detected using the antioligomer A11 and the antiamyloid fibril LOC antibodies, very little detection was observed with these antibodies using pSer26 Aβ [39]. Phosphorylation of Ser26 was found to rigidify the turn region around this modified residue leading to prevention of formation of fibrillar Aβ aggregates while stabilizing the monomeric and nontoxic soluble nonfibrillar assemblies [105].

## Conclusion

HN is known to bind Aβ protecting against its cytotoxic effects, while AChE binding to Aβ increases its aggregation and cytotoxicity [53, 74]. Previously, we found that HN and AChE can simultaneously bind Aβ in the A549 cell‐conditioned media and that HN abolishes aggregation of Aβ induced by addition of AChE [79]. We also showed that ID of HN from the media of A549 and H1299 cells increased the relative abundance of Aβ oligomer versus total Aβ, the A11‐positive prefibrillar oligomers, and to a lesser extent, the LOC‐positive fibrillar oligomers, results that correlated with diminished cell viability and increased apoptosis [79]. In this study, we set out to further understand factors affecting the interaction of Aβ with HN and AChE.

**Fig. 9:**
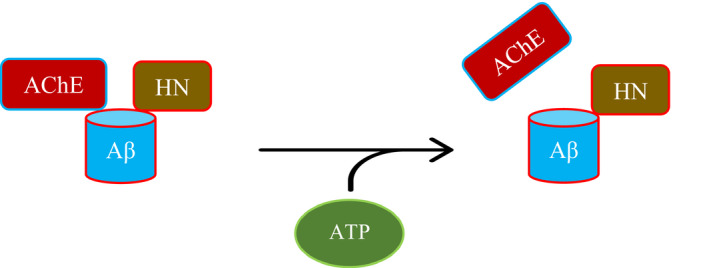
A graphic representation of the key findings of the current investigation. Both HN and AChE are able to bind Aβ in the absence of added ATP. Addition of ATP increases the binding affinity of Aβ to HN but not to AChE.

We show that the glycosaminoglycan, HS, reported earlier to interact with amino acid residues 12–18 (VHHQKLV) of Aβ40/42 peptides (Fig. 1) [51], has no effect on the binding of Aβ to either HN or AChE (Fig. 2). ATP is known to reduce misfolding and fibrillation of Aβ [42, 47]. ATP, at concentrations (200 μm) chosen to be within the extracellular physiological concentrations previously found (> 100 μm) in the lung cancer cell lines used in this study [1, 2, 3], was found to weaken interactions between AChE and Aβ (Fig. 3) but strengthens those between Aβ and HN (Fig. 4). These findings might suggest that ATP binding to Aβ promotes optimal alignment of the amino acids needed to interact with HN increasing binding affinity, while, conversely, renders the amino acid residues on Aβ important for binding AChE, less accessible (Fig. 9). Using the conditioned media of either A549 or H1299 cells, more HN was found in a complex with Aβ upon addition of ATP, while levels of AChE found in a complex with Aβ were decreased by ATP addition to A549 cell media (Fig. 5). Interaction of HN from the conditioned media of both cell lines with Aβ appears to be increased by the addition of ATP to a comparable extent, despite differences in AChE expression in the two cell lines [79, 82] likely suggesting that increased interaction of HN with Aβ by ATP is not regulated by AChE under these conditions. We also found that addition of exogenous ATP to A549 and H1299 cell‐conditioned media increased interaction of endogenous HN with Aβ and correlated with decreased binding of exogenously added HN (Fig. 6). Moreover, reduced levels of AChE were found in a complex with Aβ using A549 cell‐conditioned media with exogenously added ATP (Fig. 6). AChE might not be efficient at regulating the effects of ATP on the interaction of HN with Aβ since addition of ATP had comparable effects on the interaction of HN with Aβ using conditioned media of either A549 cells that express AChE or H1299 cells with minimal expression of the enzyme. We also show that exogenously added ATP, as high as 1 mm, had no effect on viability of either A549 or H1299 cells despite increased interactions between HN and Aβ, and, conversely, reduced binding of Aβ with AChE (Fig. 7). The lack of effect on cell viability upon addition of ATP is consistent with previous publications [3] reporting that compared to normal cells, lung cancer cells exhibit reduced cytotoxicity upon treatment with these extracellular ATP concentrations. Treatment of A549 or H1299 cells with HN‐ ID media with added ATP at either 100 µm or 1 mm concentrations had no effect on cell viability as compared to that measured in the absence of added ATP (Fig. 7). Similarly, while A549 cell viability increased ~ 1.45‐fold upon AChE ID (Fig. 7), no further effects were observed upon addition of ATP. Whether using nondepleted media, or one ID of HN or AChE, no change in the relative levels of oligomer versus total Aβ was found (Fig. 8). Factors affecting cell viability appear to depend not on additionally added ATP or its consequent effects on modulating the binding of either HN or AChE to Aβ, but rather on depleting HN or AChE from the media. Reduction of extracellular ATP levels has been previously found to correlate with increased misfolded extracellular Aβ in AD [43, 44]. While the concentration of extracellular ATP has been shown in normal tissue to be in the 1–5 μm range, in the tumor microenvironment, the concentration rises to greater than 100 μm which can induce normal cells to undergo apoptosis [1, 3]. Cancer cells including NSCLC A549 cells have been reported to release ATP and tolerate extracellular ATP concentrations that would otherwise lead to a cytotoxic response in normal cells [1]. Here, we found that treatment with exogenous ATP had no effect on cell viability under all conditions tested. Addition of higher ATP concentrations did not affect viability of cells treated with AChE‐ ID media, and there was no apparent protection against the cytotoxicity resulting from ID of HN by added ATP (Fig. 7). One possibility among many is that ATP may serve to bind Aβ decreasing its oligomerization while simultaneously serving as a substrate for extracellular kinases that might phosphorylate Aβ promoting its aggregation, resulting in the observed comparable balance of oligomer to total Aβ ratios (Fig. 8). In both cell lines, ATP might promote HN binding to Aβ enabling it to regulate access of kinases to their sites on Aβ modulating its cytotoxic effects, while AChE might provide an additional layer of regulation of Aβ phosphorylation and/or aggregation in A549 cells. Mass spectrometry analysis of extracellular fluids from cancer patients found a substantial amount of phosphorylated proteins as compared to fluids from healthy patients [106] with 84 and 32 phosphorylated sites found in the proteins from breast cancers and lung cancer samples, respectively. Whether HN or AChE plays a role in regulating Aβ phosphorylation and/or aggregation in lung cancer cells is currently a focus of research investigation in our laboratory.

## UNIPROT Accession ID

HN (Q8IVG9), AChE (C9JD78), Amyloid‐beta precursor protein (P05067).

## Conflict of interest

The authors declare no conflict of interest. The content is solely the responsibility of the authors and does not necessarily represent the official views of the National Institutes of Health.

## Author contributions

HGE designed, coordinated the study, supervised the project, and wrote the paper. SA and SD performed ELISAs, cell viability, and IDs. JD, AW, DP, and JT helped with the cell viability assays and ELISAs. DH critiqued the manuscript. JG maintained the cells and provided advice on tissue culture. All authors read and approved the final manuscript.

## Data Availability

The original data are available upon reasonable request.
